# Meteorological variables and mosquito monitoring are good predictors for infestation trends of *Aedes aegypti*, the vector of dengue, chikungunya and Zika

**DOI:** 10.1186/s13071-017-2025-8

**Published:** 2017-02-13

**Authors:** Danielle Andreza da Cruz Ferreira, Carolin Marlen Degener, Cecilia de Almeida Marques-Toledo, Maria Mercedes Bendati, Liane Oliveira Fetzer, Camila P. Teixeira, Álvaro Eduardo Eiras

**Affiliations:** 1 Departamento de Parasitologia, Instituto de Ciências Biológicas, Universidade Federal de Minas Gerais, Belo Horizonte, Brazil; 2 Programa de Computação Científica, Fiocruz, Rio de Janeiro, Brazil; 3 Ecovec Ltda., Parque Tecnológico de Belo Horizonte (BHTec) Belo Horizonte, Belo Horizonte, Brazil; 4 Departamento de Bioquímica e Imunologia, ICB, Universidade Federal de Minas Gerais, Belo Horizonte, Brazil; 5 Vigilância de Roedores e Vetores, CGVS/SMS, Porto Alegre, Brazil; 6 UNIFEMM, Sete Lagoas, Brazil

**Keywords:** *Aedes aegypti*, Dengue, Surveillance, MosquiTRAP

## Abstract

**Background:**

*Aedes aegypti* is an important vector for arboviroses and widely distributed throughout the world. Climatic factors can influence vector population dynamics and, consequently, disease transmission. The aim of this study was to characterize the temporal dynamics of an *Ae. aegypti* population and dengue cases and to investigate the relationship between meteorological variables and mosquito infestation.

**Methods:**

We monitored and analyzed the adult female *Ae. aegypti* population, the dengue-fever vector, in Porto Alegre, a subtropical city in Brazil using the MI-Dengue system (intelligent dengue monitoring). This system uses sticky traps to monitor weekly infestation indices. We fitted generalized additive models (GAM) with climate variables including precipitation, temperature and humidity, and a GAM that additionally included mosquito abundance in the previous week as an explanatory variable. Logistic regression was used to evaluate the effect of adult mosquito infestation on the probability of dengue occurrence.

**Results:**

Adult mosquito abundance was strongly seasonal, with low infestation indices during the winters and high infestation during the summers. Weekly minimum temperatures above 18 °C were strongly associated with increased mosquito abundance, whereas humidity above 75% had a negative effect on abundance. The GAM model that included adult mosquito infestation in the previous week adjusted and predicted the observed data much better than the model which included only meteorological predictor variables. Dengue was also seasonal and 98% of all cases occurred at times of high adult *Ae. aegypti* infestation. The probability of dengue occurrence increased by 25%, when the mean number of adult mosquitos caught by monitoring traps increased by 0.1 mosquitoes per week.

**Conclusions:**

The results suggest that continuous monitoring of dengue vector population allows for more reliable predictions of infestation indices. The adult mosquito infestation index was a good predictor of dengue occurrence. Weekly adult dengue vector monitoring is a helpful dengue control strategy in subtropical Brazilian cities.

**Electronic supplementary material:**

The online version of this article (doi:10.1186/s13071-017-2025-8) contains supplementary material, which is available to authorized users.

## Background

Dengue, the most important vector-borne viral disease for humans, both in terms of morbidity and economic impact, is transmitted by *Aedes* mosquitoes [1]. It is estimated that 390 million dengue infections occur every year, 96 million of which are symptomatic [2]. More than half of the world’s population is at risk of contracting the disease, mainly in urban centers of the tropics and subtropics [3]. The disease has expanded geographically in recent years, so that all four dengue virus serotypes (DENV 1–4) are now circulating in Asia, Africa and the Americas [4], and autochthonous dengue transmission has recently reached southern regions of North America and Europe [5, 6]. Urbanization, globalization, and increased international travel have contributed to this trend [7]. Predicted climate change scenarios favour a considerable increase of dengue incidence in southern Europe, especially the coastal regions [8] and an increased global distribution of the principal vector *Ae. aegypti* in areas that are currently considered to be unfavorable for this species [9].

Other important arboviruses, such as chikungunya [10] and Zika [11] can also be transmitted by *Ae. aegypti*. These two diseases present symptoms similar to dengue, however, Zika virus has recently been associated with Guillain-Barré syndrome in French Polynesia [12] and microcephaly in Brazil [13, 14].

Southern Brazil differs from other Brazilian regions by its subtropical climate, which is similar to that of southern Europe and southern United States. Therefore, a Brazilian city of subtropical climate might be a useful model for studying dengue establishment and dynamics in such areas. Systems for continuous disease and vector surveillance are important to further the understanding about the vector-disease mechanisms and disease dynamics [15, 16]. In our model city Porto Alegre, located in the Brazilian state of Rio Grande do Sul, the dengue vector and imported dengue cases were first detected in 2001 and 2002, respectively, but the first autochthonous dengue cases were confirmed only in 2010, always after the occurrence of imported cases (Coordenadoria Geral de Vigilância em Saúde, CGVS, Porto Alegre, personal communication). This is similar to the situation in some European regions, where dengue has re-emerged, also driven by the introduction of dengue-infected people (tourism and migration) and the invasion of vector mosquito species (revised by Schaffner et al. [17]). Porto Alegre city relied only on fast larval survey (LIRAa) [18] for vector surveillance, which is based on the House Index and is performed only three times per year. After dengue occurrence in 2010 and 2011, the General Health Surveillance Coordination (Coordenadoria Geral de Vigilância em Saúde, CGVS) implemented a large-scale mosquito surveillance system (intelligent dengue monitoring, MI-Dengue) that generates weekly infestation indices [15].

During the past few years, some studies have attempted to model the risk of dengue transmission and vector abundance. Due to the low occurrence of dengue in North American and European countries [19, 20], mosquito population data in these areas are often of limited temporal and spatial resolution and restricted availability. Factors that influence dengue vector abundance and disease occurrence are therefore poorly understood [17, 21]. It is well known that environmental factors influence diverse aspects of vector and virus biology by interfering with mosquito population dynamics and virus circulation [22, 23]. Furthermore climatic factors such as temperature, humidity, and rain, also affect dengue transmission (reviewed by Morin et al. [24]). However, no study has evaluated the role of these climatic factors in conjunction with vector mosquito abundance and disease surveillance in a region where seasonal autochthonous dengue transmission was recently introduced.

Therefore, the current study investigated the effects of climatic factors on female adult *Ae. aegypti* abundance and characterized the temporal profile of dengue vector population and disease incidence in a city of humid subtropical climate with a recent history of local dengue transmission. We also examined how the dengue vector density affects the probability of the occurrence of dengue infections. We used data obtained from a large-scale adult mosquito surveillance system that generates weekly infestation indices and maps in real time that is integrated with dengue disease notification in a decision support system [15, 16]. We further explored and discussed how the inclusion of continuous vector monitoring data improves the fit of vector population models. Our results have implications for dengue forecasting models and may help optimize decision making regarding vector control activities and dengue prevention measures in subtropical areas.

## Methods

### Study area

Porto Alegre (30°01′40″S, 51°13′43″W), the capital of the Brazilian State of Rio Grande do Sul, has an area of 496.68 km^2^, an estimated population of 1,409,351 inhabitants [25], and a population density of 2,837.53 inhabitants/km^2^. The city consists of about 69% natural environment and 31% urban area [26]. The climate is classified as humid subtropical according to the Köppen climate classification. This climate class is characterized by precipitation that is well distributed throughout the year [27]. In the summer (December to March), temperatures often reach 35 °C, whereas winter temperatures (June to September) range from 2 °C to 20 °C. The average annual temperature and rainfall are 19.5 °C and 1,397 mm, respectively [28].

**Fig. 1:**
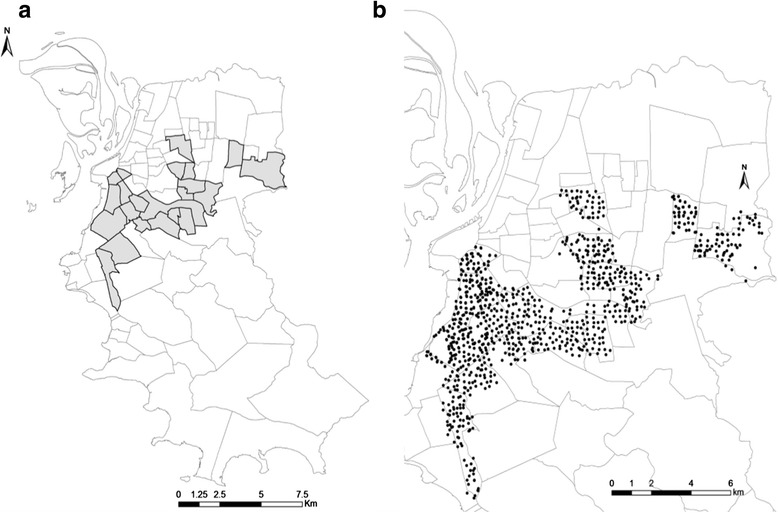
Study area. **a** Map of the municipality of Porto Alegre (Rio Grande do Sul), southern Brazil. Lines represent the borders of the neighborhoods. The neighborhoods that are monitored are represented with borders in bold. **b** Locations of MosquiTRAPs in Porto Alegre (Rio Grande do Sul)

The study was conducted in 22 of 81 neighborhoods of Porto Alegre city that are using MI-Dengue since 2012 (Fig. 1a). This area was chosen to implement the monitoring system because it was considered the most vulnerable to disease introduction and occurrence, due to high *Ae. aegypti* infestation (CGVS, Porto Alegre, personal communication).

### MI-Dengue mosquito monitoring system

MI-Dengue uses the sticky trap MosquiTRAP (Ecovec LTDA, Belo Horizonte, Brazil) to monitor the adult dengue vector population [15, 16]. The trap contains a synthetic oviposition attractant (AtrAedes) that lures gravid *Ae. aegypti*. A total of 712 sticky traps were set on fixed outdoor positions, sheltered from rain and sunlight throughout the study area (Fig. 1b), at a distance of approximately 250 m between each other. The traps were inspected weekly during 173 weeks, from September 2012 to January 2016 (epidemiological weeks 39/2012 to 2/2016). The entomological index provided by the sticky trap is the Mean Female *Ae. aegypti* Index (MFAI, the mean number of *Ae. aegypti* females per trap). After MI-Dengue was implemented in Porto Alegre city, all neighborhoods addressed their vector control based upon the MFAI index above 0.4 by source reduction.

### Meteorological data

Daily rainfall, temperature parameters (minimum, average and maximum), and average relative air humidity data were obtained from the Brazilian National Institute of Meteorology (INMET). The data were aggregated for epidemiological weeks to accumulated rainfall, and weekly average values of daily minimum, maximum and mean temperature, and minimum humidity (hereafter abbreviated as rain, Tmin, Tmax, Tmean and hum, respectively). Weekly lags of up to four weeks were included in the dataset for all meteorological variables: Tmin_t_, Tmin_t-1_, Tmin_t-2_, Tmin_t-3_, Tmin_t-4_ and hum_t_, hum_t-1_, hum_t-2_, hum_t-3_ and hum_t-4_.

### Dengue cases

The Health Secretary of Porto Alegre provided data for confirmed autochthonous dengue cases. The data were organized by epidemiological week, considering the date of first dengue symptoms.

### Statistical analysis

Prior to exploratory analysis and model fitting, the dataset was divided into a training and a test set. The training set, which was used for explanatory data analysis and model fitting, includes the first 144 weeks of the dataset (September 2012 - June 2015). The test set, which was used to compare model predictions to observational records, comprises the last 29 weeks (July 2015 - January 2016).

The relationship between meteorological variables and the number of *Ae. aegypti* females collected in MosquiTRAPs was first assessed with scatterplots (see Additional file 1: Figure S1). As there was no apparent linear relationship between mosquito catches and the explanatory variables, generalized additive models (GAM) were used to model the data. We used negative binomial models because the response variable (number of *Ae. aegypti* females collected per week) is overdispersed count data (variance = 102,880.4, mean = 332.2). Poisson models were also adjusted, but these proved to be inappropriate due to overdispersion. Since the number of weekly monitored traps was not constant, we included the logarithm of the number of monitored traps as the model offset. First, we adjusted several models with a single explanatory variable and identified the best-fitting model for each category of explanatory variables (five models [= number of lags] for each of the following: Tmin, Tmean, Tmax, hum), by comparing the Akaike Information Criterion (AIC) [29] (see Additional file 2: Table S1). We then built multiple models, adding variables in the sequence of increasing AIC of their corresponding simple models. As the three categories of temperature variables (Tmin, Tmean and Tmax) were collinear, we included only Tmin_t-4_ in a multiple model, since its corresponding simple regression model had the lowest AIC value.

The interaction between minimum temperature and humidity was also evaluated, but the model without interaction term had a lower AIC value (see Additional file 3: Table S2). The best full model with only meteorological explanatory variables is the following:

$$ \begin{array}{l} Aaefe{m}_t \sim \mathrm{Binomial}\ \mathrm{Negative}\ \left({\mu}_t,\  k\right)\\ {} \log \left({\mu}_t\right) = \log \left({N}_t\right) + \kern0.5em {f}_1\left(\mathrm{Tmi}{\mathrm{n}}_{\mathrm{t}\hbox{-} 4}\right) + {f}_2\left( hu{m_t}_{-4}\right) + {\upbeta}_{0\ }\left(\mathrm{M}1\right)\end{array} $$

where *log(N*_*t*_ *)*, the model offset, is the logarithm of the number of traps in week t (t = 1, …, 144); ƒ_1_(Tmin_t-4_) and ƒ_2_(*hum*_*t*-4_) are smooth effects of minimum temperature (lag 4) and humidity (lag 4), respectively; β_0_ is the intercept, and *k* is the dispersion parameter.

The autocorrelation plot of the M1 residues suggests an autocorrelation at lag1. In order to account for the autocorrelation and to investigate if the model’s fit improves when mosquito population data collected from the previous week are considered, we adjusted a second model, where we included a smooth effect of the mean number of female *Ae. aegypti* in the previous week (*MFAI*_*t*-1_). In this case, humidity lost significance, so that M2 is given by:

$$ \begin{array}{l} Aaefe{m}_t \sim \mathrm{Binomial}\ \mathrm{Negative}\ \left({\mu}_t,\  k\right)\\ {} \log \left({\mu}_t\right) = \log \left({N}_t\right) + \kern0.5em {f}_1\left(\mathrm{Tmi}{\mathrm{n}}_{\mathrm{t}\hbox{-} 4}\right) + {f}_2\left( MFA{I_t}_{-1}\right) + {\upbeta}_{0\kern0.37em }\left(\mathrm{M}2\right)\end{array} $$

The non-linear interaction between Tmin_t-4_ and MFAI_t-1_ was also evaluated. However, the resulting model had a higher AIC value compared to M2.

Adequacy of adjusted GAM models was evaluated through diagnostic residual plots (residuals *vs* fitted, residuals *vs* explanatory variables, autocorrelation plot, histogram and quantile-quantile plot of residuals) and by plotting the observed *versus* predicted data.

Logistic regression was used to investigate how the weekly mean female *Aedes* index and its lags of one to four weeks (MFAI_*t*_, MFAI_*t*-1_, MFAI_*t*-2_, MFAI_*t*-3_, MFAI_*t*-4_) affects the occurrence of human dengue cases. The binary response variable was the presence/absence of dengue cases in week t. The best lag was chosen based on the lowest AIC value (see Additional file 4: Table S3).

All analyses were performed in the software R, version 3.1.2 using the packages *mgcv* [30] and *MASS* [31].

## Results

### Descriptive analysis of entomological data

The sticky traps collected a total of 121,385 mosquitoes between September 2012 and January 2016. The most abundant female mosquitoes were *Ae. aegypti* (44.0%) and *Culex* spp. (38.1%), whereas *Ae. albopictus* (3.0%) was less abundant (Table 1). Male *Ae. aegypti* (0.3%) and *Ae. albopictus* (0.3%) constituted a lower proportion of the collected mosquitoes compared to *Culex* spp. males (14.2%). A mean (± standard deviation) of 0.43 ± 1.1 *Ae. aegypti* females were collected per week per trap.

**Table 1:** Descriptive statistics of mosquitoes caught in MosquiTRAPs (MQT) in Porto Alegre, Rio Grande do Sul, Brazil, between September 2012 and January 2016

Species/ stage	Total number (%)	Range (*n*)	Mean ± SD
*Aedes aegypti*			
Female	53,411 (44.0)	0–41	0.43 ± 1.10
Male	415 (0.34)	0–9	0.003 ± 0.07
*Aedes albopictus*			
Female	3,685 (3.0)	0–13	0.03 ± 0.21
Male	361 (0.3)	0–8	0.003 ± 0.07
*Culex* spp.			
Female	46,261 (38.1)	0–40	0.37 ± 1.10
Male	17,252 (14.2)	0–20	0.14 ± 0.67

*Abbreviation*: *SD* standard deviation

### Descriptive temporal analysis

**Fig. 2:**
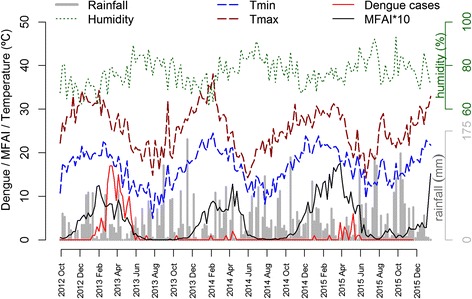
Time series of dengue cases, mean number of *Aedes aegypti* females (MFAI), precipitation, minimum and maximum temperature, and humidity in Porto Alegre, between September 2012 and January 2016

The weekly minimum and maximum temperatures ranged from 4.9 °C to 24.5 °C and from 14.1 °C to 38.0 °C, respectively, and the relative air humidity varied between 61.4 and 93%. Both temperature and humidity followed a seasonal pattern, whereas rainfall was distributed throughout the years without an apparent pattern (Fig. 2). A descriptive table by year is in Additional file 5: Table S4.

The temporal pattern of female *Ae. aegypti* abundance was seasonal during all three study years (Fig. 2). Mosquito abundance started to increase in September/October and decreased in April/May. Every year revealed a high (> 0.5) MFAI between December and April, and the highest value (1.76) was observed in the last week of March 2015. MFAI values close to zero begin to appear in June, remaining so throughout July and August. Mosquito abundance increased approximately two months after the temperature started to increase. Conversely, it began to decline simultaneously with temperature (Fig. 2). Autochthonous dengue cases also displayed a seasonal pattern. Cases occurred at times of high vector infestation and an average temperature above approximately 18 °C, and peaked when the temperature started to decrease. Most of the cases (155/181) occurred in 2013, and only 6 and 20 cases were recorded in 2014 and 2015, respectively. All autochthonous dengue cases, except 10 cases, occurred when the female *Ae. aegypti* infestation index was above 0.4 (Fig. 2).

### Relationship between mosquito abundance and meteorological variables

All time lags of temperature (Tmin, Tmean and Tmax) and humidity (hum) were significant non-linear explanatory variables of female *Ae. aegypti* abundance in univariate GAM models (*P* < 0.05) (see Additional file 2: Table S1). Rain was only significant at lag1 and approximately negatively linear. The best-fitting models of each category were *Tmean*_t-4_ *, Tmax*_t-3_ *, Tmin*_t-4_ and *hum*_t-4_. The plots of observed *versus* predicted values of the two best simple GAM models (*Tmin*_t-4_ and *hum*_t-4_) are provided in Additional file 6: Figure S2.

**Fig. 3:**
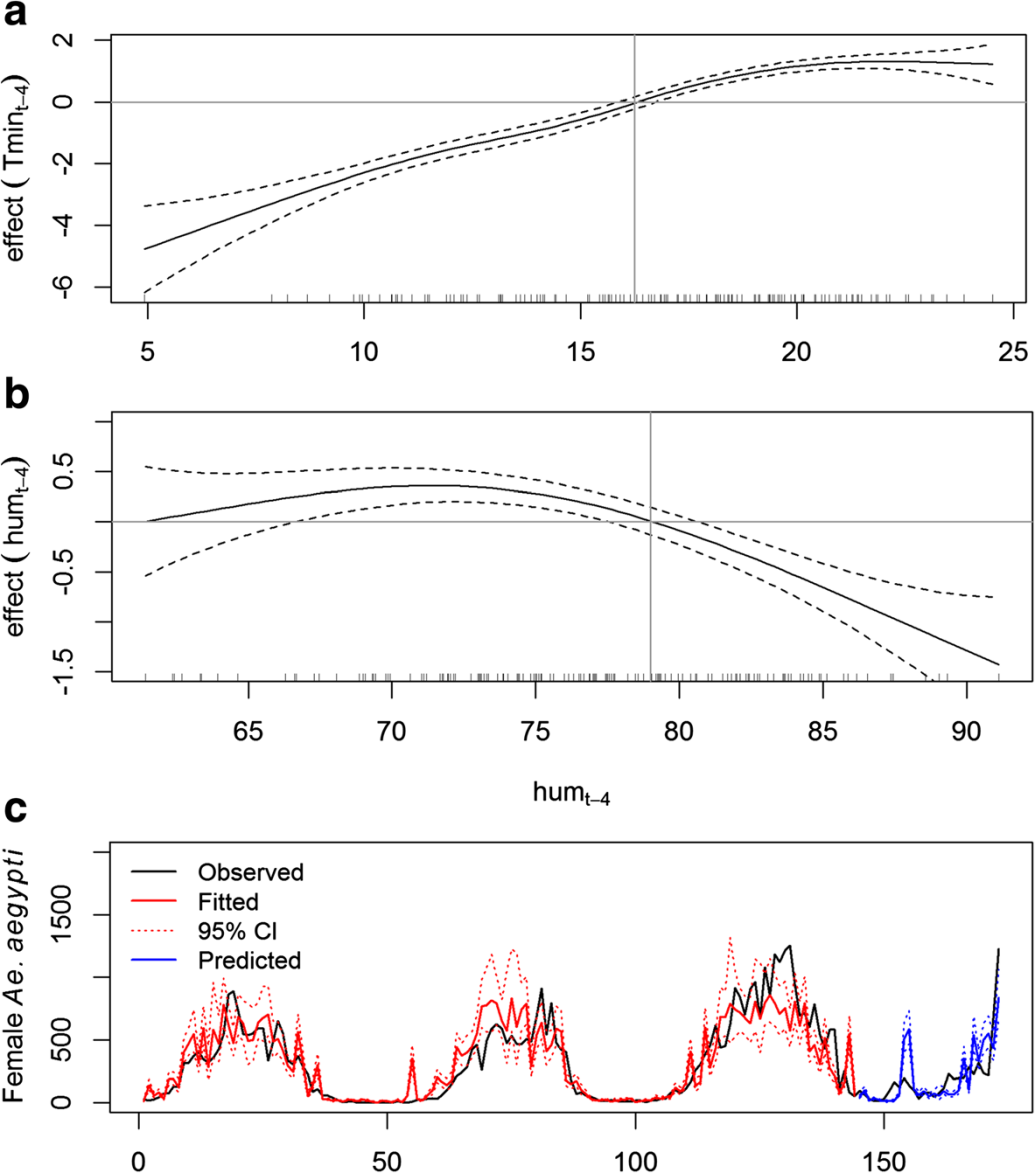
Graphical representation of the estimated results of the GAM model M1. Effects of minimum temperature lagged by four weeks (Tmin_t-4_) (**a**) and mean relative humidity lagged by four weeks (hum_t-4_) (**b**) on female *Aedes aegypti* catches in MosquiTRAPs. **c** Plot of observed *versus* fitted and predicted values

The full multiple model (M1) with meteorological variables included the non-linear predictors *Tmin*_t-4_ and *hum*_t-4_ (Table 2). The model indicates a positive relationship between mosquito abundance and minimum temperature. Above a minimum temperature of about 16 °C, the mosquito catches are above average (Fig. 3a). The effect of Tmin_t-4_ on mosquito abundance stabilizes above 19 °C (approximately horizontal line in Fig. 3a). Mosquito abundance steadily decreased when air humidity was higher than 79% (Fig. 3b). The plot of observed *versus* predicted values (Fig. 3c) indicates that the model fitted the data well, although it underestimated mosquito abundance in the third year of the study. The predicted values displayed the trend of observed data reasonably, despite the significant over- and underestimation of the mosquito population in some weeks.

**Table 2:** Output of the GAM models M1 and M2, and the logistic regression model M3. M1 and M2 are minimal adequate models to explain mosquito abundance, and M3 is the model to explain presence and absence of dengue cases

Model	Variable	Estimate	Standard Error	*χ* ^2^	*P*-value
M1	Intercept	-1.477	0.058		< 0.001
	*s*(*Tmin*_t-4_)	Smooth		352.3	< 0.001
	*s* (*hum*_t-4_)	Smooth		37.1	< 0.001
M2	Intercept	-1.590	0.038		< 0.001
	*s*(*Tmin*_t-4_)	Smooth		27.9	< 0.001
	*s*(*MAaefem*_t-1_ *)*	Smooth		296.3	< 0.001
				*z*-value	
M3	Intercept	-2.376	0.3835	-6.196	< 0.001
	MFAI_t-3_	2.298	0.499	4.599	< 0.001

**Fig. 4:**
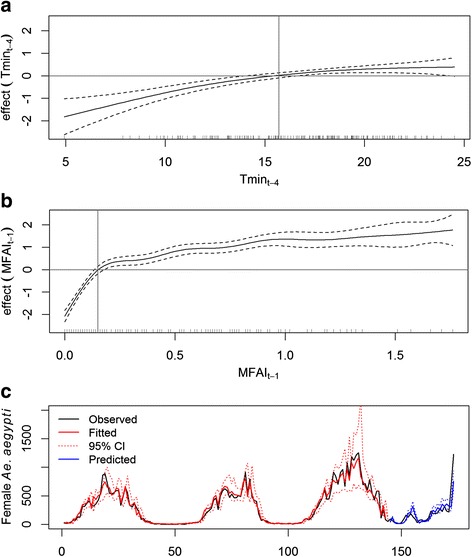
Graphic representation of the estimated results of the GAM model M2. Effects of minimum temperature lagged by four weeks (Tmin_t-4_) (**a**) and of the mean number of female *Ae. aegypti* caught in the previous week (MFAI_t-1_) (**b**) on female *Aedes aegypti* catches in MosquiTRAPs. **c** Plot of observed *versus* fitted and predicted values

The second GAM model (M2) suggests approximately the same relationship between Tmin_t-4_ and mosquito abundance, as previously described for M1 (Fig. 4a). Furthermore, the model shows that mosquito abundance increases with increasing MFAI_t-1_ values (Fig. 4b). The strongest effects are observed for MFAI_t-1_ values below 0.2, and it stabilizes when MFAI_t-1_ values reach 1.0. Figure 4c shows that predictions by the M2 model, which incorporates vector population in the previous week into the model’s mathematical equation, fits better to actual population counts (AIC = 1,628, proportion deviance explained = 87%), compared to M1, which considers only meteorological predictors (AIC = 1,735, proportion deviance explained = 74%). The predicted values closely followed the trend of observed data (Fig. 4c), confirming a superior performance of M2 in comparison to M1.

### Relationship between dengue occurrence and mean female *Aedes* index (MFAI)

**Fig. 5:**
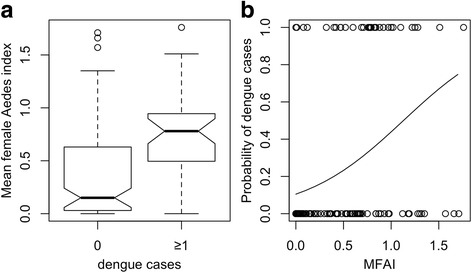
Relationship between the presence and absence of dengue cases and the mean female *Aedes* index (MFAI). **a** Box plots of MFAI conditional on dengue occurrence (0, absence of dengue cases; 1, presence of dengue cases). **b** Graph of the fitted values (solid line) obtained by the logistic regression model applied on the dengue occurrence data. The dots represent the observed values

The median MFAI values in the presence and absence of dengue cases were 0.78 and 0.15, respectively (Fig. 5a). The MFAI in all evaluated lags (MFAI_t_, MFAI_t-1_, MFAI_t-2_, MFAI_t-3_, MFAI_t-4_) significantly explained the probability of dengue occurrence (Table 2; Fig. 5b). The best lag was the MFAI of three weeks (MFAI_t-3_) (see Additional file 4: Table S3), in this lag, the probability of disease occurrence increased by 25%, when the MFAI increased by 0.1 (Table 2).

## Discussion

In this study, we found that minimum temperature, humidity and previous *Ae. aegypti* vector density are important factors affecting the temporal pattern of vector abundance in a region of subtropical humid climate in Brazil. Dengue incidence increased significantly with increasing adult female vector infestation.

Weekly mean female *Ae. aegypti* population numbers in this study followed a seasonal pattern with high infestation in the summer and very low infestation (close to zero or zero) in the winter. A similar pattern has been previously described in subtropical Argentina when using ovitraps, larval indices and larvitraps [32, 33]. Previous longitudinal studies observed intra-annual fluctuations of adult female *Ae. aegypti* collections in MosquiTRAPs in tropical Brazilian cities [34, 35]. However, in these locations, observed infestation indices rarely fell to values close to zero, reflecting a year-round dengue vector infestation. The seasonal pattern of mosquito infestation in our study was related to the seasonality of temperature and humidity. Low temperatures negatively affect dengue vector survival, which leads to a strong fall in vector population. The mean minimum daily temperature in Porto Alegre city was 2 °C during the winters, with peaks as low as 0 °C, capable of drastically decreasing adult mosquito infestation and viability of laid eggs. Even so, the mosquito population reestablishes during the summers, reaching infestation indices that are similar to and, in some weeks, even higher than in dengue-endemic tropical Brazilian cities (Ecovec LTDA, personal communication). High humidity was associated with low temperature and low mosquito infestation index. Rain was distributed throughout the whole year (characteristic of humid subtropical climate) and, therefore, was not a significant predictor of mosquito abundance.

The multiple GAM model M1 includes non-linear effects of minimum temperature and humidity, both of which lagged by four weeks. The model revealed an approximately linear, positive effect of minimum temperature on mosquito abundance up to approximately 20 °C; above this value, the effect was stable. This means that positive variations in minimum temperatures of up to 20 °C are associated with growth of the adult vector population, whilst fluctuation in minimum temperatures above 20 °C did neither increase nor decrease the mosquito population. Since temperature can also influence biting rates [36] and mosquito survival [37], these factors may affect dengue transmission. Humidity had an approximately linear negative effect on the vector population when humidity was above 79%. Humidity is associated with increased *Ae. aegypti* survival, egg development and biting rates [38–40]. The negative effect that we observed could be due to complex interactions between climatic factors. In our study, humidity was high when temperature was low. Several researchers believe that temperature is the most critical factor for the survival of *Ae. aegypti* [41–43] and this may explain the negative effect of humidity levels above 79% upon vector population.

The multiple model M2 includes a non-linear effect of minimum temperature at lag 4 and a non-linear effect of the mean number of female *Ae. aegypti* per trap with one week lag (*MFAI*_t-1_). The model indicates that the higher the number of mosquitoes in the previous week, the higher it will be the number in the next week. This effect was most prominent for MFAI values between 0 and 0.2, reflecting the sharp increase in vector abundance at the beginning of the mosquito proliferation season. The effect of MFAI stagnates at about 1, as MFAI values above 1 were rare. The results indicate that the MFAI model is more accurate and confirm that weekly vector population indices are superior for predicting vector infestation in comparison to using only meteorological predictor variables. Simões et al. [44], who analyzed mosquito infestation data in MosquiTRAPs in a tropical Brazilian city also reinforces the importance of considering previous vector population indices in such models.

The study area includes 22 neighborhoods of the city of Porto Alegre. Although it is an extensive area of the city, where the majority of the population is concentrated, it is important to be careful about the generalization of the results. In small areas there may be diverse microclimates that affect the vector population in different ways in the same interval of time. Future studies should evaluate the city’s microclimates, and evaluate for example, if the presence of heat islands affects the abundance and spatio-temporal distribution of dengue vectors and disease cases.

Another limitation of the present study is that we did not consider the effect of mosquito control interventions on the *Ae. aegypti* population and disease occurrence. Nevertheless this variable could provide information on the effectiveness of actions on the vector population, and could be analyzed in future studies.

The temporal pattern of autochthonous dengue cases was also seasonal. Dengue cases occurred approximately three months after the adult mosquito population began to increase and the peak of dengue cases in 2013 occurred soon after the peak of mosquito infestation. Previous studies analyzing the correlation between dengue incidence and adult *Ae. aegypti* population showed that high dengue vector infestation precedes high dengue incidence in tropical areas [45–47]. In all three summers in Porto Alegre city, it took two to three months until adult mosquito infestation peaked (MFAI > 0.4), which explains the observed time lag between the increase of mosquito infestation and case occurrence. The two dengue cases that occurred in June and July of 2013, at times when the MFAI had values of 0.08 and 0.00, respectively, were most likely imported cases.

The logistic regression confirmed that adult *Ae. aegypti* infestation is closely associated with subsequent dengue occurrence. Our results suggest that the weekly dengue cases increase by 25%, when the mosquito infestation increases by 0.1. Previous studies investigating the correlation between dengue cases and immature mosquito development stages (larvae and pupae) reported an absence or low correlation with dengue incidence [48, 49], indicating that the adult dengue vector monitoring is a better predictor for dengue transmission.

Due to the low total number of dengue cases, especially in 2014 and 2015, a rigorous analysis of the association between disease transmission and meteorological predictor variables was unviable. We observed, however, that cases occurred predominantly when the average temperature was above 18 °C. In subtropical Taiwan, months with temperatures above 18 °C are also associated with high risk of dengue transmission [50].

The higher number of autochthonous dengue cases in 2013, which peaked when the temperature was beginning to decrease, may suggest that besides the high vector abundance, other elements favored transmission [51]. One likely important factor was the number of imported cases in January and February (holiday season), which occurred about one week before a pronounced increase of autochthonous cases (CGVS, unpublished data). The February holiday (carnival) occurs during the dengue season in most Brazilian States and 2013 was a record epidemic year. During this time, a high number of Brazilian and foreign visitors, including from dengue-endemic cities, traveled all over Brazil. Since most dengue infections are asymptomatic [52, 53], the probability of traveling whilst being infected is relatively high, contributing to viral dissemination to other regions. Human migration patterns not only due to holidays, but also because of economic crises and wars, to name a few, appear to be important factors for estimating the likelihood importing dengue to non-endemic areas. As seen in Porto Alegre and several European and North American settings [5, 6, 54, 55], imported cases can lead to the occurrence of local transmission of diseases, when competent vector populations of *Ae. aegypti* and/or *Ae. albopictus* is present.

Another factor likely to be associated with a relatively high number of cases in 2013, when compared to 2014 and 2015 is that 2013 was the year the highest number of dengue cases in Brazil, which increased the probability of introduction of imported cases. The notification and confirmation delay of the dengue cases is the main cause of late application of control strategies. In such situations, MI-Dengue can be a good tool to detect high infestation of *Ae. aegypti* and to direct activities based on the vector index. However, no previous data from MI-Dengue monitoring was available for an area of subtropical climate, and the MFAI was not yet evaluated for this kind of scenario (Ecovec LTDA, personal communication). After three years of mosquito monitoring, we can validate the mosquito index MFAI, together with minimum temperature data, as good predictors for vector infestation and dengue incidence levels.

## Conclusions

The current study shows that minimum temperature and humidity are important meteorological variables that affect *Ae. aegypti* population dynamics. Furthermore, a model that includes data from continuous adult mosquito monitoring in addition to meteorological data adjusts and predicts the mosquito population substantially improved. We also found a strong association between female mosquito abundance and dengue case occurrence. Dengue transmission has complex dynamics, and climate factors and entomological monitoring are only a part of the disease’s determinants. Other factors, such as human movement patterns and epidemiological information (circulation serotypes, herd immunity), should also be considered for the development of reliable predictive models that estimate dengue spatio-temporal distribution in areas where it is not yet epidemic.

## Additional files

Additional file 1: Figure S1. Scatterplots of the MFAI (mean number of *Ae. aegypti* females) against each of the explanatory variables. A smoothing (LOESS) curve was added in each panel. (PDF 100 kb)
Additional file 2: Table S1. Comparison of AICs of models with variables and varying time lags. (PDF 233 kb)
Additional file 3: Table S2. Comparison of discarded models. (PDF 118 kb)
Additional file 4: Table S3. Comparison of AICs of logistic regression models with MFAI as predictor variable in varying time lags. (PDF 83 kb)
Additional file 5: Table S4. Descriptive statistics by year. * The year 2012 includes only data from September onwards, and year 2016 includes only data until January. (PDF 28 kb)
Additional file 6: Figure S2. Observed and fitted by model: **a** gam (Aaefem ~ offset(lNtraps) + s(Tmin4) + s(hum4), family = nb ()). **b** gam (Aaefem ~ offset(lNtraps) + s(hum4), family = nb ()), **c** gam (Aaefem ~ offset(lNtraps) + s(Tmin4), family = nb ()). (PDF 275 kb)

## Abbreviations

AIC: Akaike information criterion
CGVS: Coordenadoria Geral de Vigilância em Saúde
DENV: Dengue virus serotypes
GAM: Generalized additive models
INMET: Brazilian National Institute of Meteorology
LIRAa: Levantamento Rápido de Índices para *Aedes aegypti*
MFAI: Mean female *Aedes* index
MI-DENGUE: Intelligent dengue monitoring
