# Supplementary material for: Meteorological variables and mosquito monitoring are good predictors for infestation trends of Aedes aegypti, the vector of dengue, chikungunya and Zika
Source: Parasit Vectors. 2017 Feb 13;10:78. doi: 10.1186/s13071-017-2025-8 (PMC5307865; doi:10.1186/s13071-017-2025-8)
Supplement: Additional file 1: Figure S1. — Scatterplots of the MFAI (mean number of Ae. aegypti females) against each of the explanatory variables. A smoothing (LOESS) curve was added in each panel. (PDF 100 kb) [file 13071_2017_2025_MOESM1_ESM.pdf]

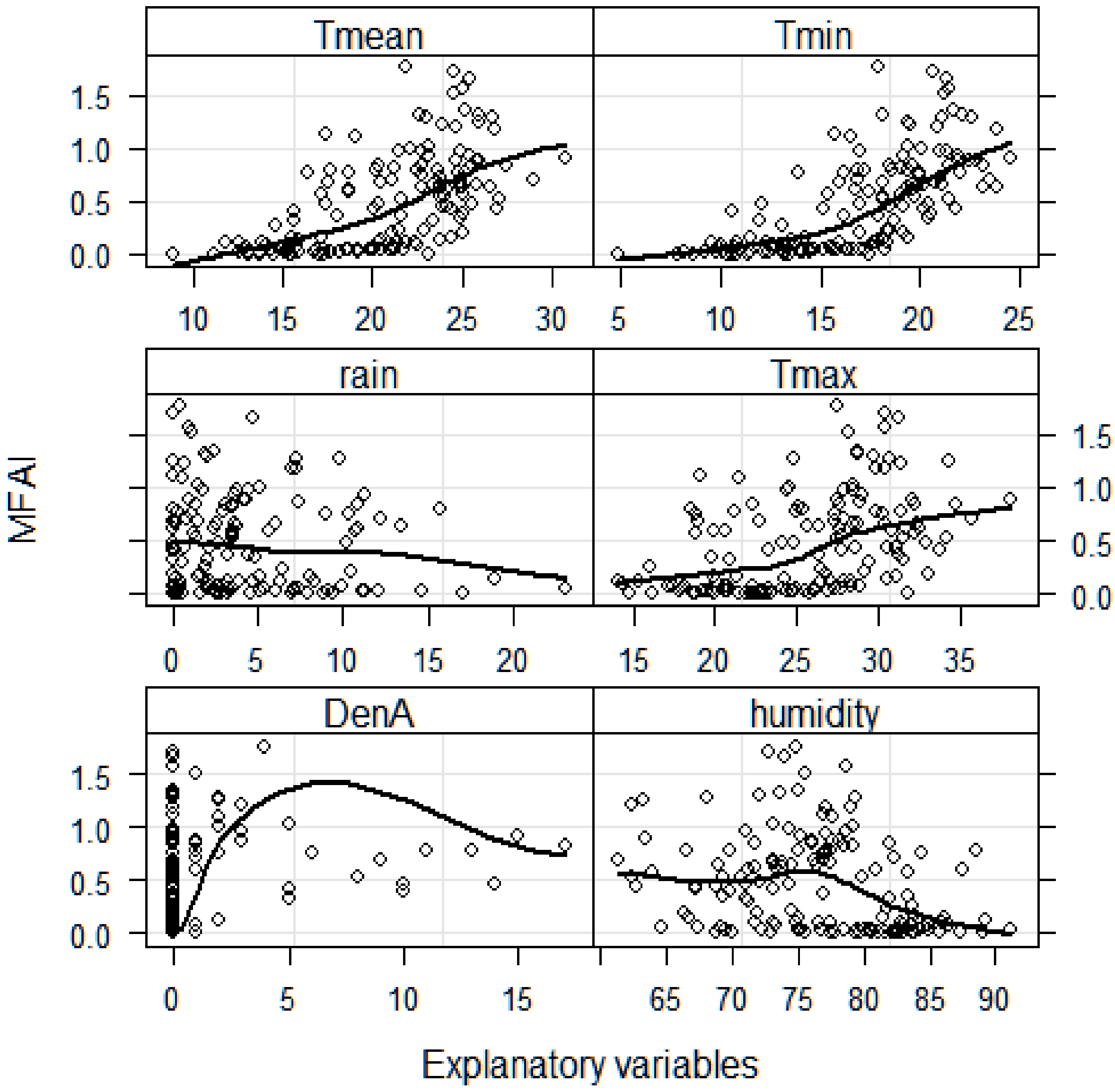

Figure S1: Scatterplots of the MFAI (mean number of *Ae. aegypti* females) against each of the explanatory variables. A smoothing (LOESS) curve was added in each panel.
