# Supplementary material for: Meteorological variables and mosquito monitoring are good predictors for infestation trends of Aedes aegypti, the vector of dengue, chikungunya and Zika
Source: Parasit Vectors. 2017 Feb 13;10:78. doi: 10.1186/s13071-017-2025-8 (PMC5307865; doi:10.1186/s13071-017-2025-8)
Supplement: Additional file 2: Table S1. — Comparison of AICs of models with variables and varying time lags. (PDF 233 kb) [file 13071_2017_2025_MOESM2_ESM.pdf]

Table S1: Comparison of AICs of models with variables and varying time lags

Formula: gam(Aaefem~offset(lNtraps)+s(variable), family=nb())

Model	Variable	Estimate	Standard Error	Chi.sq	p-value	AIC
**Models with variable explanatory Temperature minimum**						
Tmin	*Intercept*	-1.15	0.08		<0.001	1857
	s (Tmin)	Smooth		151.5	<0.001	
Tmin1	*Intercept*	-1.23	0.08		<0.001	1832
	s ($Tmin_{t-1}$)	Smooth		217.9	<0.001	
Tmin2	*Intercept*	-1.29	0.07		<0.001	1814
	s ($Tmin_{t-2}$)	Smooth		275.3	<0.001	
Tmin3	*Intercept*	-1.34	0.07		<0.001	1798
	s ($Tmin_{t-3}$)	Smooth		323.5	<0.001	
Tmin4	*Intercept*	-1.42	0.06		<0.001	1764
	s ($Tmin_{t-4}$)	Smooth		463.4	<0.001	
**Models with variable explanatory Temperature mean**						
Tmean	*Intercept*	-1.07	0.09		<0.001	1879
	s (Tmean)	Smooth		106	<0.001	
Tmean1	*Intercept*	-1.16	0.08		<0.001	1854
	s ($Tmean_{t-1}$)	Smooth		162.7	<0.001	
Tmean2	*Intercept*	-1.23	0.08		<0.001	1835
	s ($Tmean_{t-2}$)	Smooth		218.4	<0.001	
Tmean3	*Intercept*	-1.30	0.07		<0.001	1810
	s ($Tmean_{t-3}$)	Smooth		286.2	<0.001	
Tmean4	*Intercept*	-1.40	0.06		<0.001	1769
	s ($Tmean_{t-4}$)	Smooth		443.2	<0.001	

**Models with variable explanatory Temperature maximum**						
Tmax	*Intercept*	-0.87	0.10		<0.001	1916
	s (Tmax)	Smooth		26.73	<0.001	
Tmax1	*Intercept*	-0.93	0.09		<0.001	1906
	s ($Tmax_{t-1}$)	Smooth		43.18	<0.001	
Tmax2	*Intercept*	-0.96	0.09		<0.001	1900
	s ($Tmax_{t-2}$)	Smooth		54.1	<0.001	
Tmax3	*Intercept*	-1.04	0.09		<0.001	1884
	s ($Tmax_{t-3}$)	Smooth		85.12	<0.001	
Tmax4	*Intercept*	-1.14	0.08		<0.001	1860
	s ($Tmax_{t-4}$)	Smooth		140	<0.001	
**Models with variable explanatory humidity**						
Hum	*Intercept*	-0.88	0.10		<0.001	1923
	s (Hum)	Smooth		25.56	<0.001	
Hum1	*Intercept*	-0.91	0.10		<0.001	1916
	s (Hum_{t-1})	Smooth		36.32	<0.001	
Hum2	*Intercept*	-0.95	0.09		<0.001	1908
	s (Hum_{t-2})	Smooth		51.05	<0.001	
Hum3	*Intercept*	-0.980	0.09		<0.001	1901
	s (Hum_{t-3})	Smooth		65.14	<0.001	
Hum4	*Intercept*	-0.99	0.09		<0.001	1898
	s (Hum_{t-4})	Smooth		70.67	<0.001	
**Models with variable explanatory Rain**						
Rain	*Intercept*	-0.77	0.11		<0.001	1937
	s (Rain)	Smooth		2.14	0.145	
Rain1	*Intercept*	-0.78	0.11		<0.001	1934
	s ($Rain_{t-1}$)	Smooth		6.21	0.0162	

Rain2	*Intercept*	-0.77	0.11		<0.001	1937
	$s\ (Rain_{t-2})$	Smooth		4.54	0.135	
Rain3	*Intercept*	-0.77	0.11		<0.001	1938
	$s\ (Rain_{t-3})$	Smooth		3.25	0.246	
Rain4	*Intercept*	-0.76	0.11		<0.001	1938
	$s\ (Rain_{t-4})$	Smooth		2.12	0.396	
