# Supplementary material for: Meteorological variables and mosquito monitoring are good predictors for infestation trends of Aedes aegypti, the vector of dengue, chikungunya and Zika
Source: Parasit Vectors. 2017 Feb 13;10:78. doi: 10.1186/s13071-017-2025-8 (PMC5307865; doi:10.1186/s13071-017-2025-8)
Supplement: Additional file 3: Table S2. — Comparison of discarded models. (PDF 118 kb) [file 13071_2017_2025_MOESM3_ESM.pdf]

Table S2: Comparison of discarded models

**Model**	**AIC**
glm(Aaefem ~ offset(lNtraps) + $Tmin_{t-4}$ + Hum_{t-4}, family = poisson)	17337
glm(Aaefem ~ offset(lNtraps) + $Tmin_{t-4}$ + $MFAI_{t-1}$, family = poisson)	11180
glm.nb(Aaefem ~ offset(lNtraps) + $Tmin_{t-4}$ + $MFAI_{t-1}$)	1737
glm.nb(Aaefem ~ offset(lNtraps) + $Tmin_{t-4}$+ Hum_{t-4})	1763
gam(Aaefem~offset(lNtraps)+s($Tmin_{t-4}$)+s(Hum_{t-4}), family=poisson)	10159
gam(Aaefem~offset(lNtraps)+s($Tmin_{t-4}$)+s($MFAI_{t-1}$), family=poisson)	4411
gam(Aaefem~offset(lNtraps)+s($Tmin_{t-4}$, Hum_{t-4}), family=nb())	1736
gam(Aaefem~offset(lNtraps)+s($Tmin_{t-4}$, $MFAI_{t-1}$), family=nb())	1692
