# Supplementary material for: Meteorological variables and mosquito monitoring are good predictors for infestation trends of Aedes aegypti, the vector of dengue, chikungunya and Zika
Source: Parasit Vectors. 2017 Feb 13;10:78. doi: 10.1186/s13071-017-2025-8 (PMC5307865; doi:10.1186/s13071-017-2025-8)
Supplement: Additional file 4: Table S3. — Comparison of AICs of logistic regression models with MFAI as predictor variable in varying time lags. (PDF 83 kb) [file 13071_2017_2025_MOESM4_ESM.pdf]

Table S3: Comparison of AICs of logistic regression models with MFAI as predictor variable in varying time lags

Formula: glm(pDenA~MFAI, family=binomial)

**Model**	**AIC**	**P-value**
MFAI	146.7	<0.001
$MFAI_{t-1}$	147.1	<0.001
$MFAI_{t-2}$	145.7	<0.001
$MFAI_{t-3}$	137.9	<0.001
$MFAI_{t-4}$	141.4	<0.001

Abbreviations: pDenA- presence of dengue case, MFAI - mean female Aedes Index
