# Supplementary material for: Meteorological variables and mosquito monitoring are good predictors for infestation trends of Aedes aegypti, the vector of dengue, chikungunya and Zika
Source: Parasit Vectors. 2017 Feb 13;10:78. doi: 10.1186/s13071-017-2025-8 (PMC5307865; doi:10.1186/s13071-017-2025-8)
Supplement: Additional file 5: Table S4. — Descriptive statistics by year. * The year 2012 includes only data from September onwards, and year 2016 includes only data until January. (PDF 28 kb) [file 13071_2017_2025_MOESM5_ESM.pdf]

Table S4: Descriptive statistics by year.

Year		Rain	Humidity	Tmean	Tmax	Tmin	MFAI	*Aedes aegypti* (Female)
**2012***	Min	0	62.3	15.7	22.2	10.7	0.03	16
	Mean	3.5	70.1	22.7	29.3	17.9	0.27	184.7
	Max	10.8	80.6	27	34.1	21. 4	0.62	407
	Sd	3.5	5.9	2.9	3.4	2.5	0.22	149
**2013**	Min	0	62.4	8.9	14.8	4.9	0	0
	Mean	2.6	76.2	19.3	25.2	15.2	0.32	226.8
	Max	23	89.3	26.9	34.3	22.5	1.2	891
	Sd	4.8	6.3	4.4	4.9	4.1	0.36	258
**2014**	Min	0	61.4	12.5	14.2	8.2	0.01	5
	Mean	4.6	77.9	20.4	24.9	17.1	0.4	290.2
	Max	18.9	91.1	30.7	38	24.5	1.2	909
	Sd	4.4	6.3	4.5	5.2	4.2	0.35	254
**2015**	Min	0	68	11.8	14.1	9.5	0.07	51
	Mean	3.8	77.3	21.7	25.3	18.6	1	722
	Max	15.7	88.5	26.8	31.2	23.8	1.7	1252
	Sd	3.5	4.5	4	5.1	3.4	0.48	345
**2016***	Min	0.04	71.2	25.4	30.5	21.7	0.8	674
	Mean	0.25	73.4	25.8	31.7	22	1.1	950.5
	Max	0.46	75.5	26.2	32.9	22.2	1.5	1227
	Sd	0.29	3	0.6	1.7	0.37	0.4	391

* The year 2012 includes only data from September onwards, and year 2016 includes only data until January.
