# Supplementary material for: Meteorological variables and mosquito monitoring are good predictors for infestation trends of Aedes aegypti, the vector of dengue, chikungunya and Zika
Source: Parasit Vectors. 2017 Feb 13;10:78. doi: 10.1186/s13071-017-2025-8 (PMC5307865; doi:10.1186/s13071-017-2025-8)
Supplement: Additional file 6: Figure S2. — Observed and fitted by model: a gam (Aaefem ~ offset(lNtraps) + s(Tmin4) + s(hum4), family = nb ()). b gam (Aaefem ~ offset(lNtraps) + s(hum4), family = nb ()), c gam (Aaefem ~ offset(lNtraps) + s(Tmin4), family = nb ()). (PDF 275 kb) [file 13071_2017_2025_MOESM6_ESM.pdf]

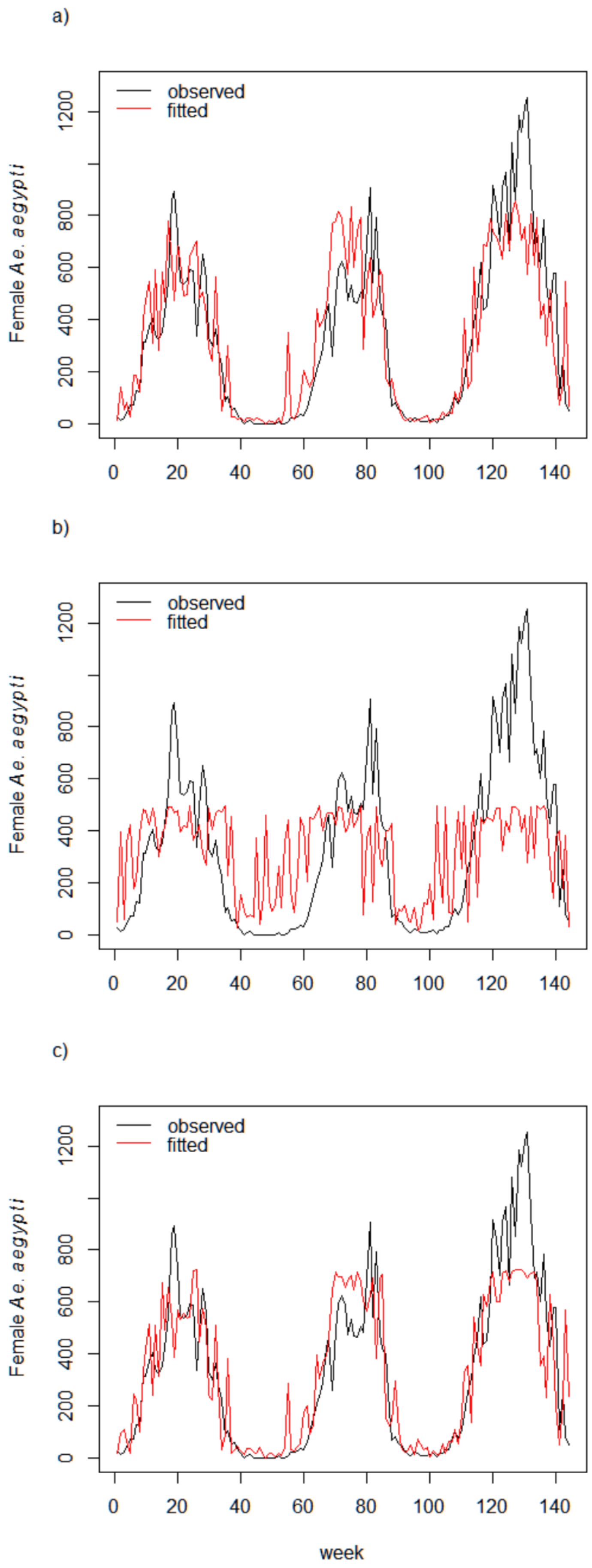

Figure S2: Observed and fitted by model: **a** gam (Aaefem~offset(lNtraps)+s(Tmin4)+s(hum4), family=nb ()), **b** gam (Aaefem~offset(lNtraps)+s(hum4), family=nb ()), **c** gam (Aaefem~offset(lNtraps)+s(Tmin4), family=nb ())
